# A Single Institution Retrospective Comparison of Two Radiotherapy Protocols for the Palliative Treatment of Canine Nasal Carcinoma

**DOI:** 10.1111/vru.70097

**Published:** 2025-10-05

**Authors:** Alison Hayes, Hannah Wong, Annette Preston, Jane Dobson

**Affiliations:** ^1^ Department of Veterinary Medicine University of Cambridge Cambridge UK

**Keywords:** adenocarcinoma, cancer, dog, intranasal, non‐lymphomatous

## Abstract

Optimal radiation protocols for canine nasal carcinoma are not established. Co‐morbidities, access, and owner compliance can influence scheduling. Between 2015 and 2022, two radiotherapy protocols were used in the palliative treatment of canine nasal carcinoma at a single institution. Group A comprised 17 cases receiving 40 Gy in ten 4 Gy fractions delivered Monday, Wednesday, and Friday. Epistaxis was present in 11/17 (65%) cases. Median survival time (MST) was 298 days (95% CI: 163.54–432.45); progression‐free survival was 173 days (95% CI: 117.87–228.12). Group B comprised 24 cases receiving 36 Gy in six 6 Gy fractions delivered Monday and Friday. Epistaxis was present in 20/24 (83%) cases. MST was 375 days (95% CI: 240.73–509.27); progression‐free survival was 243 days (95% CI: 138.42–347.58). Dogs with Adams Stage 1 disease had the longest median overall (593 days) and progression‐free survival (609 days). Four cases each received additional radiation treatment and/or toceranib at relapse. Palliative radiation therapy achieved control of clinical signs in the majority of cases, with an overall response rate of 100% (Group A) and 96% (Group B). In a multivariate Cox regression model with backwards elimination, when cases were stratified for tumor stage, neither the presence of epistaxis nor treatment (6 vs. 10 fractions) was independently associated with significant improvements in survival. Epistaxis at presentation did not appear to influence survival. These results indicate that palliative radiation therapy is highly effective in controlling clinical signs associated with nasal carcinoma. Increasing fractionation may have a limited effect on survival outcome or toxicity in the palliative setting.

## 1 Introduction

Canine nasal tumors are relatively common in dogs, comprising 1%–2% of tumors in this species [1]. Tumors of epithelial origin represent between 50% and 75% of such cases [2, 3, 4, 5]. Without treatment, the median survival time (MST) of dogs with nasal carcinomas is 95 days [6]. Progressive local invasion and destruction of the bones enclosing the nasal chamber are hallmarks of the disease and cause significant morbidity [6, 7]. Clinical signs include sneezing, epistaxis, nasal discharge, mouth breathing, dyspnea, facial swelling, facial pain, cough, decreased nasal airflow, epiphora, exophthalmos, and neurological abnormalities [6, 8]. Epistaxis is reported to occur in 67%–77% of dogs presenting with nasal carcinoma and influences survival in untreated dogs [6, 8, 9]. In one study, untreated dogs presenting with epistaxis had an MST of 88 days compared to 224 days for those without epistaxis [6]. However, in dogs undergoing radiotherapy, epistaxis at presentation was not predictive of survival [9]. Despite the potential for marked clinical signs, diagnosis is often delayed, with advanced‐stage disease often found on imaging during initial investigations [4, 10]. The modified Adams system of reporting the extent of the primary tumor (T stage) is predictive for outcome to treatment in several studies, and erosion of the cribriform plate, indicative of modified Adams Stage 4, irrespective of clinical signs, carries a worse prognosis [4, 9, 10, 11, 12 ]. Metastatic rates are between 0% and 13% at diagnosis, though rates of approximately 45% are found at necropsy [2, 3, 13, 14]. Failure to control the primary tumor burden largely influences outcome, and optimizing local disease control remains an important therapeutic goal.

The treatment of canine nasal tumors has been comprehensively reviewed [15]. Radiotherapy, cytoreductive surgery either alone or in combination with radiotherapy, photodynamic therapy [16], electrochemotherapy [17], and chemotherapy [9, 18, 19, 20] have been utilized in the treatment of the disease. Surgery following radiotherapy was shown to provide a significant increase in survival time in one study, although there was a substantial risk of developing treatment‐related complications [21]. Other studies reported no advantage when combining surgery and radiotherapy over radiotherapy alone [22, 23, 24, 25]. Despite improvements in intensive care, surgical morbidity and an inability to achieve margins of excision remain significant barriers to the use of surgery in the management of the disease [21, 26].

Radiotherapy as a single modality is currently the standard of care, but the optimal protocol has yet to be determined. The choice of radiotherapy protocol is largely dictated by access to facilities and non‐patient factors such as financial constraints, requirement for repeated general anesthesia, and hospitalization. Published results are largely small case series deploying a range of dosages, fraction sizes, and photon energies, making meaningful interpretation of treatment effect difficult. Treatment has been reviewed in the veterinary context and can be divided into two broad categories: deemed palliative intent when larger dose fractions are delivered at often weekly intervals, and definitive intent, when small (2–3 Gy) daily or alternate‐day fractions are delivered [27]. Reported MSTs for definitive intent regimes for canine nasal adenocarcinoma range from 347 to 641 days [4, 21, 28, 29, 30] and for palliative intent regimens 146 to 512 days [8, 31, 32, 33, 34, 35, 36]. Stereotactic radiotherapy (SRT) protocols, delivering large doses over a typically 1–3 day period, have partly replaced conventional radiation protocols; however, its use is still somewhat limited by the availability facilities in certain countries [37]. Progression‐free survival (PFS) and MST range from 237 to 441 and 388 to 586 days, respectively, in several small studies [12, 38, 39, 40, 41, 42].

In this study, we report the clinical response, treatment‐related adverse events, disease progression, and survival outcomes of two standardized palliative radiotherapy treatment regimens delivered within the same oncology service to two different groups of dogs diagnosed with nasal carcinoma.

## 2 Materials and Methods

This was a retrospective, observational study of dogs treated for nasal carcinoma using radiotherapy between February 2015 and May 2022. This work involved the use of nonexperimental, client‐owned animals only and followed established internationally recognized high standards of individual veterinary clinical patient care. This work was approved by the Ethics and Welfare Committee of the Department of Veterinary Medicine, University of Cambridge, May 30, 2023. Approval number CR700. Informed consent was obtained from the owner or legal custodian of all animals described in this work for the procedure(s) undertaken.

Dogs were identified by searching the radiotherapy treatment database, and cases were included on an intention‐to‐treat basis using one of two protocols. Formerly, Group A received a three‐time weekly regimen delivering 40 Gy in ten 4 Gy fractions on a Monday, Wednesday, and Friday schedule, and latterly, Group B received a twice weekly regimen delivering 36 Gy in six 6 Gy fractions on a Monday and Friday schedule. Both protocols were briefly available contemporaneously, and during this time, clients were offered both options. Other interventional treatments were not offered at this time. Three‐dimensional radiotherapy (3D RT) was delivered with a 6MV linear accelerator without the capability of a multi‐leaf collimator (MLC), intensity‐modulated radiotherapy (IMRT), or SRT delivery (Varian Clinac 2100 IX DMX, Varian, Palo Alto, CA, USA). Equipment underwent daily in‐house quality assurance and dose output checks in addition to monthly dosimetry calibration by an external medical physicist. Dogs were excluded if they did not have a cytologically or histologically confirmed diagnosis of a sinonasal carcinoma or if they had previously been treated with surgery, radiotherapy, chemotherapy, or targeted therapy. Prior treatment with antibiotics, steroids, and nonsteroidal anti‐inflammatory drugs (NSAIDs) was permitted. Staging procedures were performed at the discretion of the attending clinician and included imaging of the thorax by three‐view radiography or CT and fine needle aspirate cytology or histological examination of medial retropharyngeal and/or mandibular lymph nodes. Other diagnostic procedures comprised a complete blood count, chemistry panel, urinalysis, and abdominal imaging and were performed on a case‐by‐case basis.

Following diagnostic assessment, dogs underwent contrast‐enhanced CT of the head under general anesthesia for radiation planning. Slice thickness of the CT was 1 mm, and windowing selection was L:35/W:350. MRI was not utilized. Forward planning was performed using the Addenbrooke's Radiation Planning System (ARPS v1.91).^1^ A single radiation oncologist (JMD) prescribed and approved treatment plans. Treatment planning was standardized and utilized the Batho algorithm for homogeneity correction. Organs at risk (OAR) were defined as the brain and eyes, and sparing of OAR was prioritized over target coverage. The clinical target volume (CTV) was defined as the gross tumor volume (GTV) plus a 5 mm margin, where possible, based on 3D CT images. The CTV to PTV expansion was to the bony nasal casing, where there was no bone invasion, and was expanded rostrally and caudally by 1 cm. The treatment planning aim was to deliver at least 95% of the prescribed dose to the PTV with a maximum dose of 107%. Treatment was evaluated manually by visual inspection of isodose lines and dose‐volume histograms, including the OAR, and was delivered in 1–4 one beams as determined by the treatment plan. A 0.5–1 cm tissue‐equivalent bolus was used to achieve an adequate dose at the tumor surface on an individual basis. Treatments were delivered under general anesthesia according to previously published reports, and repeatable positioning was achieved using individual bite blocks and vacuum bags with the dogs in sternal recumbency [43]. Position verification using imaging was not available.

Data retrieved from medical records included age, sex, neuter status, presence or absence of epistaxis, time to diagnosis (defined as the time from the onset of clinical signs attributable to the tumor to histological or cytological diagnosis), staging procedures (radiography or CT thorax and lymph node interrogation), presence of metastasis, method of diagnosis (histology vs. cytology), time to treatment (defined as the time from diagnosis to the first radiotherapy session), duration of treatment protocol, treatment adjustments or delays, and any subsequent therapeutic interventions. The extent of local disease was categorized according to the modified Adams tumor staging criteria (Table 1), determined retrospectively from imaging reports by ECVDI‐certified veterinary radiologists.

**TABLE 1:** Modified Adams staging system for canine intranasal tumors [10].

Stage	Description
**I**	Confined to one nasal passage, paranasal, and frontal sinus, no bone involvement beyond turbinate
**II**	Any bone involvement with no evidence of involvement of orbit, subcutaneous/submucosal tissue
**III**	Any involvement of the orbit and/or nasopharyngeal and/or subcutaneous/submucosal tissue
**IV**	Involvement of the cribriform plate/invasion of the brain

Response to treatment was assessed by owner history and clinical examination at the primary care practice or treatment center at 2‐ and 8–10 weeks following the end of treatment. An absence of clinical signs associated with the nasal tumor, such as a notable improvement in nasal airflow and resolution of previous clinical signs such as sneezing, epistaxis, nasal discharge, mouth breathing, dyspnea, epiphora, facial swelling, facial pain, cough, exophthalmos, or neurological abnormalities was defined as a complete clinical response (cCR). Where clinical signs remained, but an overall improvement was noted, this was deemed a partial clinical response (cPR). A worsening of clinical signs or the development of new clinical signs associated with the nasal tumor was deemed to be progressive clinical disease (cPD). Where patient records provided incomplete follow‐up, primary care practices were contacted to retrieve clinical status, including signs of recurrence and survival data.

Radiation toxicity was graded according to VRTOG morbidity criteria [44]. Where toxicity grade was not assigned by the attending clinician, this was retrospectively evaluated from clinical notes and images. Acute toxicity was defined as toxicity seen from the time of first treatment until the scheduled 8–10 weeks posttreatment examination. Toxicity after this time was deemed to be late toxicity. When clinical signs of rhinitis were seen, supportive care in the form of anti‐inflammatory and antibiotic treatment was offered. Cases nonresponsive to medical management were offered repeat CT imaging and biopsy. Progressive disease was defined as clinical signs of rhinitis that were nonresponsive to medical management; recurrence of original clinical signs; or imaging findings of tumor regrowth with or without cytohistological confirmation. PFS was defined as the period from commencement of radiotherapy to progressive disease or death. Cases were censored if the dog was alive and there was no evidence of progressive disease at the time of data analysis, or the dog had been lost to follow‐up with no prior suspicion of progressive disease. Overall survival (OS) was calculated from the date of histological or cytological diagnosis to the date of death. Cases were censored if the animal was alive at the time of data analysis or if the dog had been lost to follow‐up.

Two palliative rescue treatments were permitted. When delivered, a second radiation treatment was patient‐dependent and not standardized. Tyrosine kinase inhibitor therapy was permitted at first relapse but was not routinely given following the initial course of radiotherapy. Dogs treated with rescue therapy as described were included in the OS analysis, and the date of first recurrence was used for PFS analysis.

Survival times were calculated using the Kaplan–Meier product‐limit method and were assessed by log‐rank tests. Proportional hazard assumptions were confirmed. Variance inflation factors were calculated, and collinearity diagnostics did not detect multicollinearity. To control for the effect of Adams stage, changing the baseline hazard of death or tumor progression, the model was stratified by Adams stage when determining the prognostic effect of treatment protocol and the presence of epistaxis. The optimized model was created using backwards elimination using multivariate Cox regression with α equal to the Akaike information criterion, 0.157 [45]. The hazard ratios, the B coefficients, and *p* values generated by the multivariate Cox regression were created using 1000 bootstrap samples. Statistically significant differences were defined as *p* < 0.05. Statistical calculations were performed using SPSS Statistics (Version 29, IBM Corp., Armonk, NY, USA) and GraphPad Prism (Version 10, GraphPad Software, La Jolla, CA, USA). Statistical analysis was performed by (HW), who received training as part of an advanced degree.

## 3 Results

The time from onset of clinical signs to diagnosis, time from diagnosis to starting treatment, and the duration of treatment for both groups are shown in Table 2. Radiation dose statistics and OAR dose data for available cases in both groups are shown in Tables 3a and 3b. Repeat clinical assessments at the treatment center are summarized in Table 4; four dogs in each group underwent repeat CT during the follow‐up period. At the time of data analysis, 3 dogs were alive, 35 dogs were dead, and 3 dogs were lost to follow‐up for both survival and disease progression.

**TABLE 2:** Groups A and B time to diagnosis, treatment, and treatment duration.

	Group A			Group B		
	Median	Interquartile range	Range	Median	Interquartile range	Range
Clinical signs to diagnosis	103	53–175	12–382	62	29.5–154.5	1–421
Diagnosis to start treatment	18	16–24	6–35	24.5	16.75–33.25	4–45
Length of treatment	21	21–21	7–28	17.5	17–18	7–21

**TABLE 3a:** Radiation dose statistics, first‐line therapy Groups A and B.

	Group A		Group B	
	40 Gy (4 Gy × 10) 15/17 cases		36 Gy (6 Gy × 6) 20/24 cases	
Total dose	Median	Range	Median	Range
D98% GTV (Gy)	35.74	2.19–42.65	32.37	10.20–34.69
D50% GTV (Gy)	39.96	38.22–41.26	35.75	34.72–40.15
D2% GTV (Gy)	41.96	40.78–45.61	37.45	35.46–38.97
Homogeneity index (GTV)	0.19	0.00–1.01	0.14	0.07–0.77
D98% PTV (Gy)	29.60	1.16–37.62	27.13	7.09–33.73
D50% PTV (Gy)	39.28	36.88–41.13	35.43	34.30–36.50
D2% PTV (Gy)	41.98	40.71–47.18	37.63	36.55–39.43
Homogeneity index (PTV)	0.3	0.08–1.05	0.25	0.11–0.86
**Planning**				
Single field	3		1	
Two beams	8		9	
Three beams	1		10	
Four beams	3		0	
No eye block	11		7	
Left eye block	2		6	
Right eye block	0		3	
Bilateral eye block	2		4	
Brain block	1		1	

*Note*: Homogeneity index (D2%–D98%)/D50%.

**TABLE 3b:** Summary dose data to organs at risk (OAR).

Group A 40 Gy (4 Gy × 10) (15/17 cases)						
	Left eye		Right eye		Brain	
Dose (%)	Median	Range	Median	Range	Median	Range
Min	2.0	0.6–33.0	3.10	0.7–53.6	0.3	0.0–5.8
Max	55.9	5.2–106.1	24.7	3.2–110.2	99.9	7.6–116.3
Median	8.3	2.5–47.8	22.8	1.7–101.5	7.6	0.6–31.7

Group B 36 Gy (6 Gy × 6) (20/24 cases)						
	Left eye		Right eye		Brain	
Dose (%)	Median	Range	Median	Range	Median	Range
Min	2.5	0.0–41.4	2.7	0.1–32.7	0.1	0.00–4.2
Max	89.9	4.4–101.3	80.0	6.0–102.7	100.0	0.00–109.5
Median	17.6	0.9–68.9	14.0	2.0–57.2	6.6	0.00–34.8

**TABLE 4:** Treatment center duration of clinical follow‐up.

	Group A		Group B	
Hospital examinations	Cases	%	Cases	%
6–8 weeks	3	18	6	25
2–9 months	5	29	8	33.3
9–18 months	6	35	3	12.5
Not examined	3	18	7	29.2
Total	17	100	24	100

**FIGURE 1:**
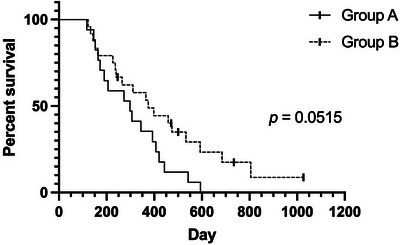
Kaplan–Meier of overall survival of canine nasal carcinoma cases treated by either protocol A (40 Gy in 4 Gy fractions, three sessions per week) or protocol B (36 Gy in 6 Gy fractions, two sessions per week). Log rank *p* = 0.0515. Symbols indicate censored cases. This relationship was further investigated using multivariate analysis, which also supported the conclusion that the protocol did not impact overall survival.

**FIGURE 2:**
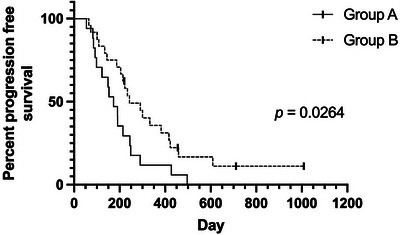
Kaplan–Meier of progression‐free survival of canine nasal carcinoma cases treated by either protocol A (40 Gy in 4 Gy fractions, three sessions per week) or protocol B (36 Gy in 6 Gy fractions, two sessions per week). Log rank *p* = 0.0264. Symbols indicate censored cases. This relationship was further investigated using multivariate analysis, and the impact of the protocol upon progression‐free survival did not persist; therefore, we conclude that protocol choice did not impact progression‐free survival in this study population.

Group A comprised 17 cases treated between 2015 and 2020: 7 females (5 neutered) and 10 males (7 neutered). Breeds included were cross‐breed (4), Labrador Retriever (4), Border Collie (2), Golden Retriever (2), and one each of Wheaten Terrier, German Shepherd Dog, Bedlington Terrier, Spitz, and Samoyed. Median age was 10.0 years, range 6y8m–14y0m. Epistaxis was present at diagnosis in 11/17 (65%) cases. Modified Adams stage at presentation was Stage 1 (0 cases), Stage 2 (8 cases), Stage 3 (4 cases), and Stage 4 (5 cases). Clinical response data were available in 16/17 cases; there was a cCR in 6/16 (37.5%) cases and a cPR in 10/16 (62.5%). The overall clinical response (cOR) was 100%. Overall, MST was 298 days (95% CI 163.54–432.45), and PFS was 173 days (95% CI: 117.87–228.12) (Figures 1 and 2).

Group B comprised 24 cases treated between 2018 and 2022, 11 females (9 neutered) and 13 neutered males. Breeds included were cross‐breed (5), Labrador Retriever (3), Border Collie (2), Staffordshire Bull Terrier (3), Cocker Spaniel (3), and one each of Golden Retriever, Lurcher, Greyhound, English Springer Spaniel, Weimaraner, Poodle, Beagle, and Grand Basset Griffon Vendeen. Median age was 10y8m, range 4y0m–13y2m. Epistaxis was present at diagnosis in 20/24 (83%) cases. Modified Adams stage at presentation was Stage 1 (5 cases), Stage 2 (3 cases), Stage 3 (5 cases), and Stage 4 (11 cases). Clinical response was available in 24/24 cases: There was a cCR in 12/24 (50%) cases, cPR in 11/24 (46%) cases, and cPD in 1/24 (4%) cases. The cOR was 96%. Overall, MST was 375 days (95% CI: 240.73–509.27), and PFS was 243 days (95% CI: 138.42–347.58) (Figures 1 and 2).

**FIGURE 3:**
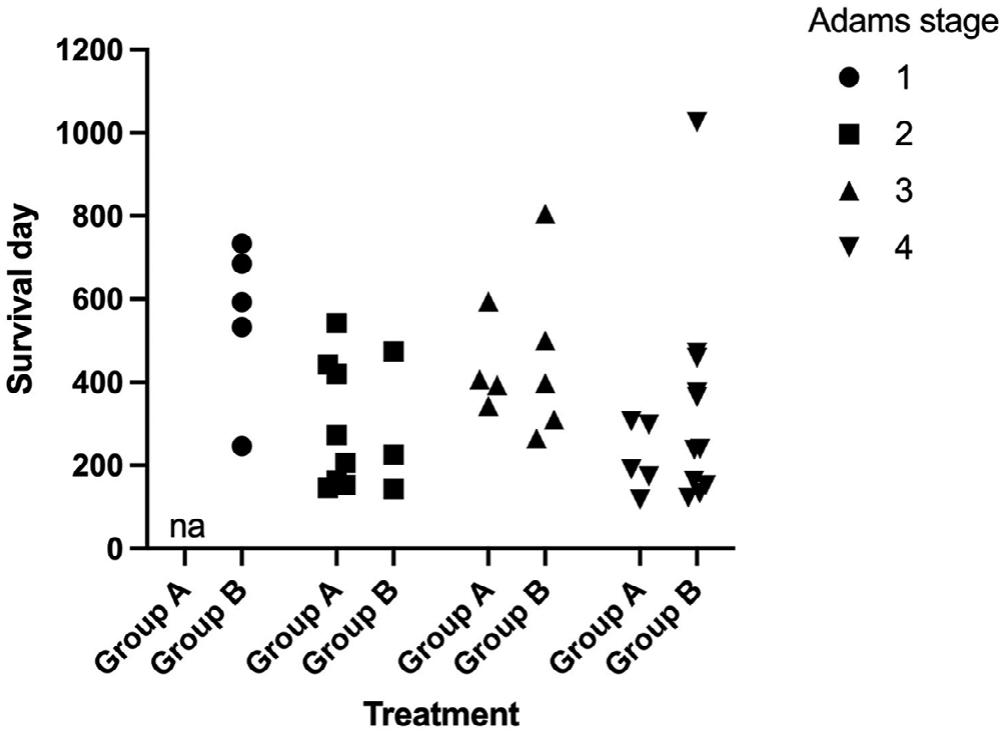
Overall survival of cases of canine nasal carcinoma grouped by treatment received and stratified by Adams tumor stage.

**FIGURE 4:**
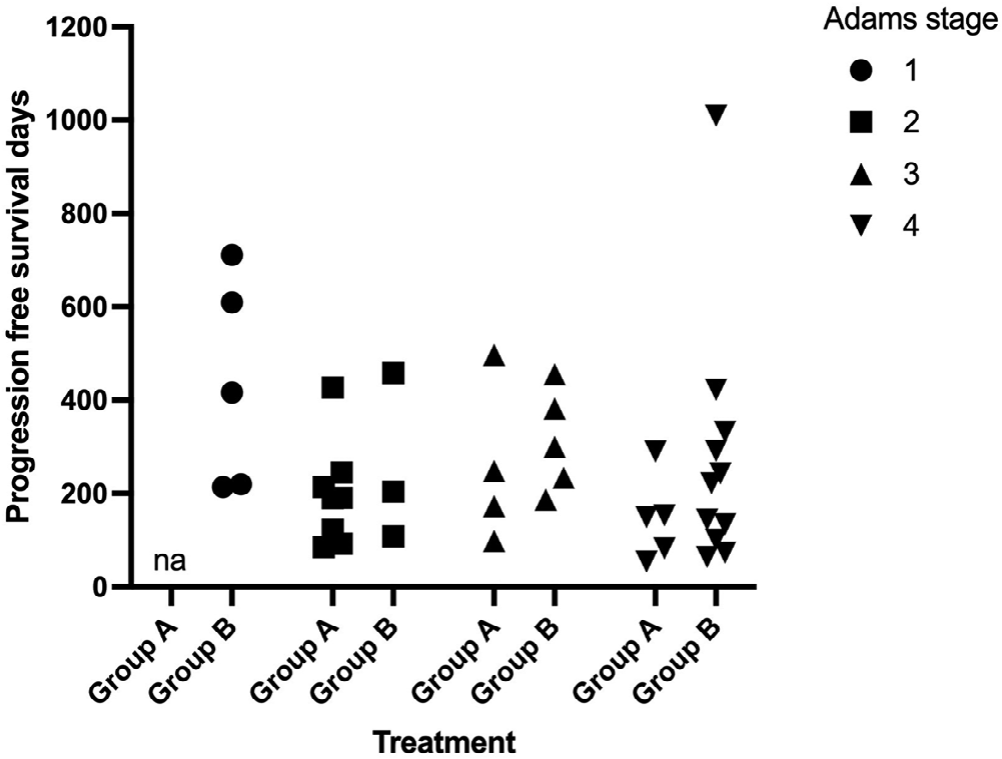
Progression‐free survival of cases of canine nasal carcinoma grouped by treatment received and stratified by Adams tumor stage.

When stratifying the cases according to the Adams stage, Stage 1 cases demonstrated the longest overall MST (593 days), and Stages 2 and 4 had the shortest overall MSTs (226 and 237 days, respectively). Stage 3 cases had an intermediate overall MST (398 days) (Table 5). For PFS, a similar pattern was present; Stage 1 cases demonstrated the longest PFS (609 days); Stages 2 and 4 had shorter PFS (191 and 149 days, respectively), and Stage 3 cases had an intermediate PFS (248 days) (Table 6). To control for an effect of Adams stage, changing the baseline hazard of death or tumor progression, the model was stratified by Adams stage when determining the prognostic effect of treatment and the presence of epistaxis. In a multivariate Cox regression model with backwards elimination, when cases were stratified for Adams stage, neither the presence of epistaxis nor treatment (6 vs. 10 fractions) was independently associated with significant improvements in OS and PFS (Tables 7 and 8). The differences between groups based on Adams stage, and a lack of observable difference in OS and PFS of the treatment cohorts while stratified by Adams stage, can be observed in Figures 3 and 4.

**TABLE 5:** Overall survival (not stratified for stage) of cases grouped by different clinical variables calculated by Kaplan–Meier log‐rank analysis.

Variable	Group	Number	MST days	95% CI	*p* value (KM log rank)
Treatment	Group A	17	298	163.545–432.455	0.0515
	Group B	24	375	240.729–509.271	
Adams stage	1	5	593	444.04–741.96	0.038*
	2	11	226	108.395–343.605	
	3	9	398	383.391–412.609	
	4	16	237	139–335	
Epistaxis	Yes	31	375	274.52–455.48	0.881
	No	10	273	161.435–384.565	

Abbreviation: MST, median survival time.

* Statistically significant differences were defined as *p* < 0.05

**TABLE 6:** Progression‐free survival (not stratified for stage) of cases grouped by different clinical variables, calculated by Kaplan–Meier log‐rank analysis.

Variable	Group	Number	MPFS days	95% CI	*p* value (KM log rank)
Treatment	Group A	17	173	117.873–228.127	0.0264
	Group B	24	243	138.417–347.583	
Adams stage	1	5	609	281.296–936.704	0.101
	2	11	191.000	102.527–279.472	
	3	9	248	207.095–288.905	
	4	16	149	131.360–166.640	
Epistaxis	Yes	31	243	169.545–307.455	0.968
	no	10	191	169.307–212.693	

Abbreviation: MPFS, median progression‐free survival.

**TABLE 7:** Backwards elimination to produce an optimized multivariate model for the effect of clinical variables on overall survival.

Multivariate regression with backwards elimination stratified for Adams stage				Base model			Final model	
Parameter	Groups	No	MST	HR	*p* ^a^	Backwards elimination	HR	*p*
Treatment	Group A	17	298	0.613	0.247		0.672	0.293
	Group B	24	375					
Epistaxis	Absent	10	273	1.584	0.342		Eliminated	
	Present	31	375					

Abbreviation: MST, median survival time.

^a^ Based on 1000 bootstrap samples.

**TABLE 8:** Backwards elimination to produce an optimized multivariate model for the effect of clinical variables on progression‐free survival.

Multivariate regression with backwards elimination stratified for Adams stage				Base model			Final model	
Parameter	Groups	No	MPFS	HR	*p* ^a^	Backwards elimination	HR	*p*
Treatment	Group A	17	173	0.528	0.122		0.541	0.105
	Group B	24	243					
Epistaxis	Absent	10	191	1.231	0.653		Eliminated	
	Present	31	243					

Abbreviation: MPFS, median progression‐free survival.

^a^ Based on 1000 bootstrap samples.

### 3.1 Additional Treatment

At the time of progressive disease, four cases received additional treatment, comprising 12–24 Gy delivered using variable protocols determined by the discretion of the prescribing radiation oncologist. Three cases were in Group B: These comprised 24 Gy delivered in once weekly 6 Gy fractions; 16 Gy delivered in twice weekly 8 Gy fractions; and 12 Gy delivered in once weekly 6 Gy fractions in one case. The fourth dog was in Group A and received 20 Gy in four 5 Gy fractions on a Monday, Wednesday, and Friday basis. Four cases with progression of disease received toceranib (Palladia, Zoetis UK Ltd., Surrey, UK). Two cases were in Group A: PFS/overall MST occurred at 150/299 and 191/544 days. Two cases were in group B: PFS/overall MST occurred at 312/376 and 609/685 days.

### 3.2 Toxicity

Toxicity was recorded for dogs undergoing a primary course of radiation therapy. Most toxicity events were Grade 1 in both groups (Table 9). In Group A, toxicity grading was recorded contemporaneously for 8 events and retrospectively for 19. Data were unavailable for the assessment of toxicity in five cases (one in the acute period and four in the late period). In Group B, toxicity grading was recorded contemporaneously for 4 events and retrospectively for 28 events. Data were unavailable for the assessment of toxicity in four cases in the late toxicity period. When early toxicity occurred, mucositis was recorded most frequently in both groups. Ocular toxicity comprised 4 of 27 recorded events in Group A, and 8 of 32 recorded events in Group B, which included 4 grade 2 acute events in this group. Early or late toxicity involving the central nervous system and late bone‐associated toxicity were not recorded in either group.

**TABLE 9:** Groups A and B: toxicity events (VRTOG morbidity score).

Group A	Toxicity events	Total treated	Affected cases	% cases treated with toxicity
**10 fractions**	27	17	15	88
		**Grade 1**	**Grade 2**	**Grade 3**
Acute (18)	**Mucositis**	12	0	0
(1.05/case treated)	**Cutaneous**	1	2	0
	**Ocular**	2	1	0
Late (9)	**Cutaneous**	8	0	0
(0.53/case treated)	**Ocular**	0	1	0

Group B	Toxicity events	Total treated	Affected cases	% cases treated with toxicity
**6 fractions**	32	24	18	75
		**Grade 1**	**Grade 2**	**Grade 3**
Acute (20)	**Mucositis**	7	2	1
(0.83/case treated)	**Cutaneous**	3	0	0
	**Ocular**	3	4	0
Late (12)	**Cutaneous**	11	0	0
(0.5/case treated)	**Ocular**	1	0	0

## 4 Discussion

The goal of palliative radiotherapy is to increase the quality of life of the patient without onerous treatment‐related toxicity [46, 47]. A paucity of treatment centers and the requirement for general anesthesia in veterinary patients has resulted in radiotherapy protocols delivered over longer time intervals and at higher doses than may seem optimal for curative intent treatment. Any improvements in survival and protection against late‐occurring radiation effects achieved by definitive treatment regimens utilizing small doses of radiation delivered more frequently must be balanced by the cost, travel, or hospitalization and a greater tendency to acute toxicities and consequent treatment‐associated morbidity [27]. With consideration of these contextual factors, it is through this lens that we consider the protocols reported here.

In this study, we report the outcomes of two palliative intent protocols delivered at a single institution. Treatment was delivered to Group B cases within six outpatient visits, compared to ten such visits for Group A cases, resulting in a considerable saving in terms of cost and client resources. Both protocols achieved similarly high levels of cOR and survival, without significant differences in outcome between protocols when cases were stratified for tumor stage. The OS times achieved in both protocols are comparable with previous studies reporting OS in both the definitive (overall MST 347–641 days) [4, 21, 28, 29, 30] and palliative settings (overall MST 146–512 days) [8, 31, 32, 33, 34, 35, 36].

Several studies have shown that the Adams stage is a key predictor of treatment outcomes [4, 9, 10, 11, 12]. Consistent with these findings, the current study also reports that dogs with Adams Stage 1 disease had the longest median overall (593 days) and progression‐free survival (609 days). There was no statistical difference in PFS and OS between the two groups. The tendency to longer PFS and OS in Group B likely correlates with the larger number of early stage clinical presentations, as measured by the distribution of Adams stages between the cohorts. The differing distribution of Adams stage data at presentation could suggest improved detection and/or awareness by primary care practices, with cases being referred for investigation and treatment at an earlier disease stage in the later treated Group B cases.

Despite limitations in reporting toxicity in this retrospective study, more acute toxicities were recorded in Group A (1.05/cases treated) compared to Group B (0.83/cases treated), yet both treatment protocols were well tolerated, with the majority of toxicity events recorded as Grade 1 and resulting in few schedule changes. When considering late toxicities, these were equally reported between groups and were largely limited to Grade 1 cutaneous effects. Due to restrictions in place during the COVID‐19 pandemic, slightly fewer dogs in Group B underwent follow‐up examinations at the treatment center, placing more reliance on primary care clinical records. This may have contributed to some underreporting of toxicity in Group B.

In this study, epistaxis was present at diagnosis in 67% of Group A dogs and 83% of Group B dogs (overall 31/41, 76%) and was broadly consistent with previous reports [6, 8, 9]. It is interesting to speculate whether the higher number of Group B dogs presenting with epistaxis, a clinical sign perhaps least well tolerated or overlooked by owners, may also have contributed to the shorter time to treatment due to earlier presentation of Group B dogs for investigation. However, palliative radiotherapy with either protocol was a very effective method of controlling clinical signs with an overall response rate of 100% in Group A and 96% in Group B. Epistaxis at presentation was not predictive of outcome in this study, though control of epistaxis was not determined specifically as an endpoint.

In common with veterinary oncology studies, endpoint bias due to owner‐determined euthanasia remains a relevant confounding factor in the study of nasal tumors in dogs [10]. Further studies are needed to better understand factors that contribute to owner decision‐making around euthanasia, though we speculate that epistaxis is likely to be poorly tolerated by owners and that good control of epistaxis would be likely to contribute to improved survival outcomes.

There were inevitable limitations of this small, non‐randomized, retrospective study, which was not designed to directly compare two protocols. A small number of cases were additionally treated at relapse on an individual basis with either an additional radiation treatment or were medically managed with toceranib. Further treatments were not offered in all cases, and a decision to further treat was often determined by owner‐related factors. Recent publications indicate that canine nasal carcinoma is responsive to medical management with toceranib and could benefit dogs in the palliative setting as a single modality treatment or in combination with radiotherapy but was not routinely offered to dogs in this study [20, 48, 49]. Few studies have been published documenting the efficacy and toxicity of re‐irradiation for nasal tumors in dogs, yet this is understood to contribute to further disease control [50]. Although there were a small number of cases managed in this way in both groups, the impact on OS cannot be fully determined. The use of supportive care, which included an NSAID either strategically or long‐term, was permitted in this study and could have affected the outcome due to perceived improvements in quality of life, or as a possible radiosensitizer [51, 52]. Due to variations in drug, duration of dosing, and dosing in relation to radiation therapy, these data were not reported, although most dogs treated received at least one course of an NSAID during the study period. Incomplete records and the necessity for some retrospective allocation of toxicity grading were weaknesses of the study. However, acute high‐grade toxicity was unlikely to remain uncaptured due to the lack of radiation treatment delays and the absence of owner‐reported concerns in the acute follow‐up period. Tumor volume and histological subtype have been found to have prognostic relevance and were not analyzed for survival outcome in this study [53]. Advanced imaging was not routinely performed posttreatment, nor always achieved at clinical relapse. Consequently, the case outcome was largely determined by clinical assessment and may have resulted in an overestimation or underestimation of progression‐free survival in this study. Additionally, patterns of local treatment failure with respect to the treatment field could not be determined [54].

Palliative treatment options such as those described herein can be achieved with minimal treatment‐associated morbidity. Furthermore, costs associated with care delivery may be minimized by judicious choice of dosing regimen when assurances on tolerability and comparable extension of life expectancy can be provided. Based on survival outcomes and toxicity, these results suggest that there may be no clear benefit in increasing fractionation for palliative intent protocols in the management of canine nasal carcinoma and that traditionally hypo‐fractionated protocols that are less time‐intensive, less expensive, well tolerated, and improve quality of life, albeit for shorter durations than definitive approaches, may suit the goals of many pet owners.

## Author Contributions

Conception and design: Alison Hayes, Hannah Wong, and Jane Dobson. Acquisition of data: Alison Hayes and Annette Preston. Analysis and interpretation of data: Alison Hayes and Hannah Wong. Drafting the article: Alison Hayes, Hannah Wong, and Jane Dobson. Reviewing article for intellectual content: Alison Hayes, Hannah Wong, Annette Preston, and Jane Dobson. Final approval of the completed article: Alison Hayes, Hannah Wong, Annette Preston, and Jane Dobson. Agreement to be accountable for all aspects of the work in ensuring that questions related to the accuracy or integrity of any part of the work are appropriately investigated and resolved: Alison Hayes, Hannah Wong, Annette Preston, and Jane Dobson.

## Disclosure

This research has not been previously published. The research was presented at the European Society of Veterinary Oncology annual congress, Bucharest, May 23–25, 2024. Adjustments were not made following the utility of a checklist.

## Conflicts of Interest

The authors declare no conflicts of interest.

## Data Availability

Data can be accessed at www.repository.cam.ac.uk.

## Endnotes

1 Software written by Dr S Thomas, Dept. Medical Physics, Addenbrookes Hospital, Cambridge (tested in accordance with IPEMB‐Institute of Physics and Engineering in Medicine and Biology) 1996. Report 68. A guide to commissioning and quality of treatment planning systems. Ed J. E. Shaw (York, IPEM)
